# Fusarium Head Blight in Argentina, a Profile of Produced Mycotoxins and a Biocontrol Strategy in Barley During Micro-Malting Process

**DOI:** 10.3390/toxins17010039

**Published:** 2025-01-15

**Authors:** María Silvina Alaniz-Zanon, Marianela Bossa, Lorenzo Antonio Rosales Cavaglieri, Juan Manuel Palazzini, Michael Sulyok, Sofía Noemí Chulze, María Laura Chiotta

**Affiliations:** 1 Instituto de Investigación en Micología y Micotoxicología (IMICO), Consejo Nacional de Investigaciones Científicas y Técnicas (CONICET)-Universidad Nacional de Río Cuarto (UNRC), Ruta Nacional 36 Km 601, Río Cuarto 5800, Argentina; mbossa@exa.unrc.edu.ar (M.B.); jpalazzini@exa.unrc.edu.ar (J.M.P.); schulze@exa.unrc.edu.ar (S.N.C.); 2 Departamento de Microbiología e Inmunología, Facultad de Ciencias Exactas, Físico-Químicas y Naturales, Universidad Nacional de Río Cuarto (UNRC), Ruta Nacional 36 Km 601, Río Cuarto 5800, Argentina; lorenzorosales3@gmail.com; 3 Christian Doppler Laboratory for Mycotoxin Research, Department IFA-Tulln, University of Natural Resources and Applied Life Sciences, Vienna, Konrad Lorenzstr. 20, A-3430 Tulln, Austria; michael.sulyok@boku.ac.at

**Keywords:** Fusarium Head Blight, biocontrol, mycotoxins, barley, micro-malting

## Abstract

Barley (*Hordeum vulgare* L.) is the second winter crop in Argentina. In the national market, grains are mainly destined to produce malt for beer manufacture. *Fusarium* species are common, causing Fusarium Head Blight (FHB) in barley, which generates yield and quality losses, as well as mycotoxin occurrence. The aims of this study were to determine (a) the incidence of the main species causing FHB in different locations of the barley-growing region of Argentina, (b) their ability to produce mycotoxins, and (c) the levels of deoxynivalenol (DON) and nivalenol (NIV) natural occurrence in grains at the harvest stage. Additionally, a strain of *Bacillus velezensis* was studied as a biocontrol agent in order to control *F. graminearum* sensu stricto and mycotoxin accumulation during the malting process, with the final objective being to reduce DON contamination in the beer manufacture chain. *Fusarium graminearum* ss was the most prevalent species causing FHB, with *Fusarium poae* being less distributed. Both species produced several mycotoxins, including NX-2 and NX-3, which is the first report of their production by strains isolated from barley in Argentina. Deoxynivalenol contamination was found in 95% of barley grains during the 2016 harvest season (mean: 0.4 mg/kg), while NIV contamination was present in 29% of samples (mean: 0.49 mg/kg). In the 2017 harvest season, 53.6% of grains were contaminated with DON (mean: 0.42 mg/kg), and 21% with NIV (mean: 0.8 mg/kg). Quantification of *F. graminearum* ss by real-time PCR during the micro-malting process showed that application of the biocontrol agent before the germination stage was the most effective treatment, with a 45% reduction in fungal DNA levels. Reduction in DON contamination (69.3–100%) in artificially infected grains with *F. graminearum* ss, was also observed. The present work contributes to the knowledge of FHB in Argentina and to the development of a strategy to control this disease and mycotoxin contamination in barley, promoting at the same time food security.

## 1. Introduction

Barley (*Hordeum vulgare* L.) is an important cereal that grows worldwide and most of it is used for forage, feed and food, malt and beer production, and distilled beverages [1,2]. In Argentina, barley grains are predominantly utilized by the brewing industry. Approximately 75% of the country’s barley production is exported, while the remaining 25% is allocated to the domestic market for malt production in beer manufacturing [3].

Cereals such as wheat and barley can be affected by a disease known as Fusarium Head Blight (FHB), which is one of the most worrying illnesses for these crops since it is associated with mycotoxin production. More than 17 *Fusarium* species have been related to FHB [4]. The species *Fusarium graminearum* sensu stricto (*F. graminearum* ss) and *F. poae* have been described as the main etiological agents in several countries around the world, including Argentina [5,6,7,8,9,10,11]. Those species are producers of different kinds of mycotoxins comprising type B trichothecenes, such as nivalenol (NIV), deoxynivalenol (DON), and their acetylated derivatives [12]. *Fusarium graminearum* ss also produces zearalenone (ZEN) and culmorin, being relatively recurrent in several cereals in co-occurrence with DON [1,13,14]. Zearalenone can be metabolized into many other derivatives such as zearalanone (ZAN), α-zearalanol (α-ZAL), ß-zearalanol (ß-ZAL), α-zearalenol (α-ZEL), and ß-zearalenol (ß-ZEL) [15]. Culmorin, a tricyclic sesquiterpene diol, is an emerging mycotoxin that has a synergistic effect with DON [16]. Conversely, it has been demonstrated that *F. poae* in addition to producing NIV, is also a producer of diacetoxyscirpenol (DAS) (type A trichothecene) and beauvericin (BEA) [17].

As regards type A trichothecenes, NX-2 and NX-3 have been identified in rice and characterized. NX-2 (3α-acetoxy-7α,15-dihydroxy-12,13-epoxytrichothec-9-ene) is similar to 3-ADON (3-acetyl-deoxynivalenol), although it lacks the keto group at C-8. This mycotoxin, as well as 3-ADON, has less toxicity than NX-3, which inhibits protein biosynthesis to almost the same extent as DON [18]. The acetylated trichothecenes 3-ADON, 15-ADON, 4-ANIV, 4,15-diANIV, and NX-2 produced in culture are mostly converted to their deacetylated forms, DON, NIV, and NX-3 in planta [16]. Monoacetoxyscirpenol (MAS) is another type A trichothecene reported in barley samples from different countries, including malting barley samples. However, more studies on monitoring its prevalence in these products are necessary. T-2 toxin is frequently detected in cereals like barley, especially in cooler and humid regions. Different metabolic pathways can transform T-2 toxin into metabolites such as T-2 triol, T-2 tetraol, and neosolaniol (NEO) [19].

The occurrence of mycotoxins in food and feed ingredients is considered a critical food safety issue, mainly in economically developing regions. Acute ingestion of these trichothecenes through consumption of contaminated cereals has been proven in the case of DON and ZAN to cause various toxicological effects [20]. Due to those detrimental effects, international legislation exists to regulate food contamination. The European Commission established maximum limits for main *Fusarium* mycotoxins in cereals and sub-products destined for human consumption [21]. Currently, this legislation includes acceptable limits of from 200 to 1750 µg/kg for DON contamination in cereals. However, since usually NIV contamination is close to DON and they are detected simultaneously in different food matrices [22], there is no regulation for NIV, based on the assumption that the established regulation for DON will prevent NIV exposure. Regarding ZAN, acceptable levels vary from 20 and 200 µg/kg in cereals. Maximum guidance levels for other toxins have not been defined, but several studies show the importance of detecting these non-regulated and emerging mycotoxins due to high occurrence patterns and concentration levels in grains, food, and feed.

After water, barley is the most important raw material in beer production. Several malt types with particular characteristics are achieved by adjusting different parameters during the malting process [2]. Barley malting is a complex biological process in which barley germination leads to hydrolytic enzyme synthesis as well as kernel structure degradation [23]. The microbial communities related to malting are developed in three stages: before harvest, during storage, and during the malting process [24]. In this last step, a favorable ecosystem for microbial growth is generated, considering nutrient, moisture, and temperature conditions. Therefore, these microorganisms significantly influence yield and final malt quality [25,26,27]. Previous studies have revealed that *Fusarium* species are able to grow and produce DON during the processes of steeping, germination, and kilning. The development of fungal species and the synthesis of their metabolites affect malt and beer [28,29]. The immediate effects of pre-harvest infection over beer manufacture are the reduction in seed germination rate during malting, the production of abundant foam (known as gushing), and/or color or flavor changes in the final product [30,31].

Currently, there is a great variety of physical, chemical, and biological tools to reduce the symptoms and yield impact on crops affected by plant pathogenic and/or toxigenic fungi and the subsequent sub-product contamination [32]. The indiscriminate use of agrochemicals results in food, soil, water, and environmental contamination [33]. In this context, several strategies based on new, safe, biodegradable, and economically feasible approaches have been developed. A plan of action called Agenda 2030 has been designed and implemented at a global level in order to follow the objective of zero hunger, focused on food security, among other 16 sustainable development goals [34,35]. Additionally, the projected global population increase will demand greater availability of safe and innocuous food, requiring efficient mycotoxin management. Biological control may be one of the few options that show the potential to preserve health [36].

Several strategies have been developed in order to control *F. graminearum* ss as the causal agent of FHB in wheat and barley, and responsible for contamination with several mycotoxins of concern [37,38,39]. However, unsatisfactory results after using these strategies have led to the development and implementation of additional tools to reduce both disease and mycotoxin accumulation. In this sense, the application of biological control agents is a strategy that can be included in an integrated pest management and offers an effective and eco-friendly alternative to reduce FHB. Several studies using biocontrol agents against *F. graminearum* ss have been conducted in wheat and barley, although most of these studies are focused on wheat [40,41,42,43,44,45,46,47,48,49,50,51,52].

Related specifically to barley, Alaniz-Zanon et al. [53] evaluated the biocontrol effect of *Bacillus velezensis* RC218 on the incidence and severity of FHB in the field trials and showed that it was more efficient against *F. graminearum* ss and DON accumulation than against *F. poae* and NIV occurrence. However, biological control strategies of *Fusarium* species and mycotoxin accumulation in barley during the micro-malting process have not yet been deeply developed. In this context, the present work aimed to determine (a) the incidence of the main species causing FHB in different locations of the barley-growing region of Argentina, (b) the ability of those species to produce mycotoxins, and (c) the levels of DON and NIV natural occurrence in grains at the harvest stage. Furthermore, we aimed to evaluate if biological control based on a strain of *B. velezensis* would be efficient during the malting process to control *F. graminearum* ss and to reduce mycotoxin contamination. The development of this kind of technological tool may contribute to improving the safety of beer manufactured from barley-contaminated grains.

## 2. Results

### 2.1. Fusarium Species Incidence in Barley Grains

*Fusarium* isolation frequencies varied among the evaluated seasons and regions (*p*-value < 0.05). During the 2016 harvest season, the highest incidence was observed in the Victoria and Carcarañá regions, while during the 2017 harvest season, this was detected in the Ferré and Bigand regions. As regards barley varieties, no differences were observed in 2016, but during 2017, the most infected variety was Voyager (mean = 99%), followed by Shakira (mean = 93%), Andreia (mean = 92%), MP2122 (mean = 91%), Montoya (mean = 90%), MP1012 (mean = 89%), and Danielle (mean = 79%). Only the infection percentages of the Voyager and Danielle varieties were statistically different (*p*-value < 0.05).

**Figure 1:**
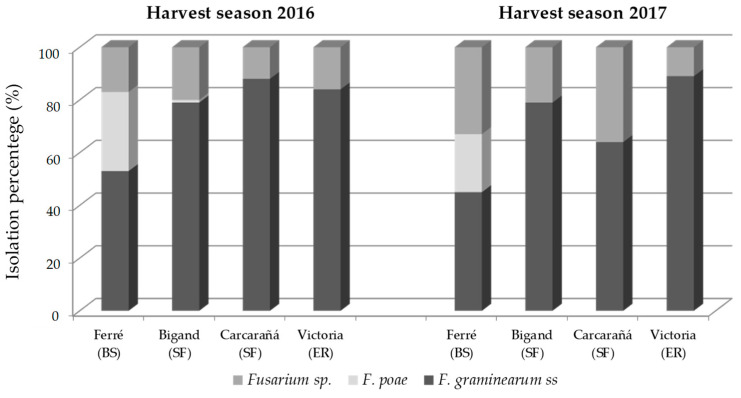
*Fusarium* species identified in different localities from barley-growing regions during 2016 and 2017 harvest seasons. BS: Buenos Aires, SF: Santa Fe, and ER: Entre Ríos provinces.

Out of 572 *Fusarium* isolates from barley grains, 76% were morphologically identified as species belonging to *F. graminearum* species complex and 12% to *F. poae*. All *F. graminearum* species complex strains were identified as *F. graminearum* ss when molecular analysis was performed. This species was dominant in all barley-growing regions while *F. poae* was relevant only in the Ferré region (*p* < 0.05) (Figure 1). *Fusarium graminearum* ss was isolated in higher percentages in the Carcarañá and Victoria regions during the 2016 harvest season, and in the Victoria region during the 2017 harvest season.

During the 2016 harvest season, *F. graminearum* ss and *F. poae* were isolated from all barley varieties, with isolation percentages ranging from 57 to 88%, and from 2 to 24%, respectively. During the 2017 harvest season, *F. graminearum* ss was observed in high levels in all barley varieties, ranging from 52% to 93%. *Fusarium poae* was isolated from the Montoya, Andreia, Danielle, and MP2122 varieties, representing 48%, 36%, 27%, and 9%, respectively.

### 2.2. Fusarium graminearum Sensu Stricto and Fusarium poae Toxigenic Profiles

Table 1 and Table 2 show the toxigenic profiles of *F. graminearum* ss and *F. poae* strains, respectively. *Fusarium graminearum* ss strains showed the DON, 15-ADON, 3-ADON, and NIV chemotypes. The percentage of toxigenic strains varied according to the year and the variety from which they were isolated. The highest mycotoxin levels were produced by the strains isolated from the Montoya variety during the 2017 season. As regards type A trichothecenes, the production of NX-2 and NX-3 was detected. From the total evaluated strains, 82% produced NX-2 and 54.5%, NX-3. Zearalenone, ZEN-sulphate, α-ZEN, and β-ZEN production was also observed. Other metabolites such as sambucinol, hydroxyculmorins, culmorin, aurofusarin, butenolid, and gibepyrone D characterized the *F. graminearum* toxigenic profile.

*Fusarium poae* was able to produce NIV simultaneously with other mycotoxins. Ninety-one percent of the total strains were NIV producers and the toxicogenic ability varied according to the evaluated season. The strains isolated during the 2016 harvest season showed higher levels of toxin production (mean: 81.3 mg/kg) than the strains isolated during the 2017 harvest season (mean: 49.6 mg/kg). Type A trichothecenes and depsipeptides production such as DAS, NEO, MAS, T-2 tetraol, and BEA was detected, representing 63.2%, 78.9%, 71%, 39.5%, and 100% of the isolated strains, respectively. Considering other metabolites, strains were able to biosynthesize aurofusarin (86.8%), butenolide (36.8%), and gybepyrone D (76.3%).

### 2.3. Mycotoxin Incidence in Barley Grains

Although all varieties showed high percentages of *F. graminearum* ss contamination, the DON levels detected in barley grains were low, ranging from 0.1 to 1.7 mg/kg (Table 3). *Fusarium poae* was isolated in higher percentages in the Montoya (37.8%), Danielle (18.9%), and Andreia (13.5%) varieties and NIV contamination ranged from 0.1 to 1.0 mg/kg. The highest NIV level was detected in the Andreia variety.

As regards harvest seasons, a higher percentage of barley samples (95%) were contaminated with DON during the 2016 season in comparison with the 2017 season (53.6%), but the mean levels detected were similar. Conversely, the percentages of contaminated samples with NIV were similar in both harvest seasons (29% in 2016 and 21% in 2017) but the contamination levels with this mycotoxin were higher during the 2017 season (0.37–0.62 mg/kg in 2016 vs. 0.70–0.89 mg/kg in 2017).

### 2.4. Biocontrol During Micro-Malting 

**Figure 2:**
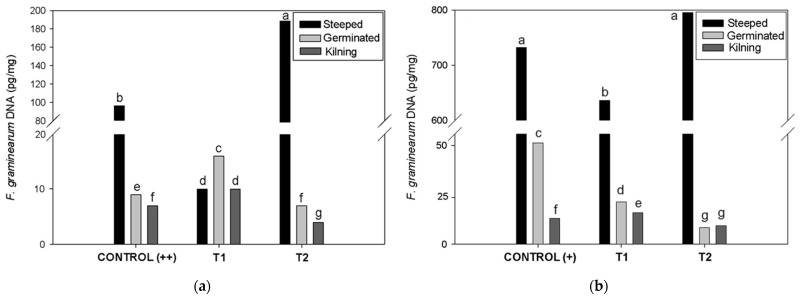
*Fusarium graminearum* ss DNA quantification by real-time PCR at the end of steeping, germination, and kilning stages. (**a**) Control (++) assay: barley grain infected artificially with *Fusarium graminearum* ss in the field trials and (**b**) Control (+) assay: naturally contaminated barley grains with *Fusarium graminearum* ss. Treatment 1 (T_1_): *Bacillus velezensis* RC218 application previous to the beginning of the steeping stage; Treatment (T_2_): application of biocontrol prior to the germination stage. Sampling stages from both assays: at the end of steeping, germination, and kilning. Values not sharing a common letter are significantly different (*p*-value < 0.05).

Evaluation of DNA dynamics of *F. graminearum* ss by real-time PCR showed a decrease in the fungal levels during the micro-malting process, in both controls (Figure 2). Contamination levels with *F. graminearum* ss significantly decreased at the end of the micro-malting process, when the biocontrol agent was applied before the steeping stage. However, a higher effectiveness was observed at the end of the process, when *B. velezensis* RC218 was applied before the germination stage.

**Figure 3:**
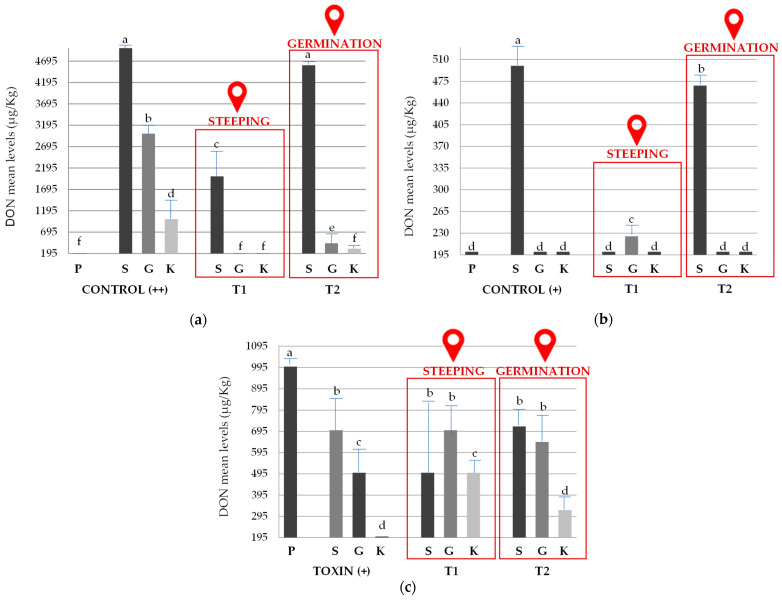
Assays on deoxynivalenol occurrence during different micro-malting stages. (**a**) Control (++) assay: barley grain inoculated artificially with *Fusarium graminearum* in the field. (**b**) Control (+) assay: naturally contaminated barley grains with *Fusarium graminearum* ss. (**c**) Toxin (+) assay: naturally barley grains contaminated with high DON levels. Treatment 1 (T_1_): *Bacillus velezensis* RC218 application previous to the beginning of the steeping stage; Treatment (T_2_): application of biocontrol prior to the germination stage. Pre-malting (P), steeping (S), germination (G), and kilning (K) stages of the sampling. Values not sharing a common letter are significantly different (*p*-value < 0.05).

Deoxynivalenol accumulation increased at the end of the steeping stage of the micro-malting but decreased at the end of the germination and kilning stages in both *F. graminearum* ss controls (Control ++ and Control +) (Figure 3). In relation to toxin accumulation on grains previously contaminated with DON (Toxin +), a different result was observed since DON levels decreased at the end of the steeping stage and continued to decrease in the rest of the stages. When the biocontrol agent was applied prior to steeping and germination stages (T_1_ and T_2_, respectively), DON levels decreased in both experiments with fungal inoculum-contaminated grains, being significantly lower than in treatment without *B. velezensis* RC218 application. In the toxin experiment, although a decrease in DON levels was observed at the end of the process, no significant differences were found when compared with the control (Toxin +).

Table 4 shows the quality parameters of the samples in each treatment. These results demonstrate that the biocontrol agent did not produce significant changes in the grain quality.

## 3. Discussion

A high incidence of *Fusarium* species in the Ferré, Bigand, Carcarañá, and Victoria regions was detected. The geographical area corresponding to these regions is known as temperate pampas (or humid subtropical) and their climatic conditions frequently favor fungal growth.

Within *Fusarium* species causing FHB in cereal grains, *F. graminearum* ss and *F. poae* are the most common species isolated from barley in South America [6,54,55,56]. In the present study, *F. graminearum* ss was isolated from all regions evaluated during the 2016 and 2017 seasons, whereas *F. poae* infection was restricted only to some regions. The year did not show relevant differences in relation to the incidence of both species; however, the region had a more important impact on their isolation. In the Ferré region (Buenos Aires province), *F. poae* was isolated in higher percentages than in the Bigan, Carcarañá, and Victoria regions. These results are consistent with previous studies conducted in Argentina, which reported a high frequency of *Fusarium poae* in barley samples harvested from a nearby area in the Buenos Aires province [56,57]. The prevalence of *F. poae* in a specific geographic region could be related to crop rotation, management practices, and mainly, to the environmental factors, such as temperature and relative humidity that play a key role in their survival. Unlike *F. graminearum* ss, *F. poae* grows and develops in arid conditions, and its distribution is more limited [58]. The climatic conditions in Buenos Aires province, being drier compared to the other regions (Santa Fe and Entre Ríos provinces) with higher humidity, may further contribute to the higher prevalence of *F. poae* in this region. In this context, the climate change scenario could increase the incidence and severity of *F. poae* in crops as a consequence of drought periods that could be more conducive for their infection [5,58,59,60]. In this sense, continuous monitoring of these species in different regions and years will be crucial to better understand the factors influencing their distribution and to predict potential future risks.

Barley genotypes were important in relation to the susceptibility to *F. poae* infection and NIV contamination. The Montoya and Andreia varieties were the most susceptible genotypes. In contrast, *F. graminearum* ss was isolated in all evaluated cultivars. Stenglein et al. [61] evaluated FHB symptoms on several barley genotypes inoculated with *F. poae* and concluded that FHB incidence and disease severity were genotype-dependent. In contrast with the results shown in the present work, Nogueira et al. [57], who studied some of the genotypes used in this study, did not find significant differences between genotypes and the percentages of *F. poae* isolation and/or NIV contamination.

Within the *F. graminearum* species complex, the 15-ADON chemotype has been predominant in strains isolated from barley and wheat in Argentina, Brazil, and Uruguay [6,62,63,64,65,66,67,68,69]. Our study provides a new insight into the chemotype of the *F. graminearum* ss, reporting that 91% of the strains produced simultaneously both 3-ADON and 15-ADON. Similar results have been reported by Castañares et al. [55], who observed that DON, 15-ADON, and 3-ADON were simultaneously produced by most of the *F. graminearum* strains isolated from barley. Recently, these strains were studied in ecophysiology assays, and the pattern of toxin production was influenced by temperature and a_w_, showing differences in DON, 15-ADON, and 3-ADON production [70,71].

*Fusarium graminearum* ss strains producing NX were previously investigated in wheat and barley in South American countries such as Argentina [64], Brazil [72], and Uruguay [54], but no strain was isolated in these regions. In the present study, NX-2 and NX-3 production was detected for the first time by strains isolated from barley in Argentina. The NX mycotoxins are important since their production not only affects *F. graminearum* spread but also initial infection on wheat heads [73]. Moreover, the NX toxins could provide an advantage to the fungus by circumventing glutathione-mediated detoxification in plants [18]. As previously mentioned, global warming is now a widely acknowledged fact and the changes in the agricultural factors will influence the structures of *Fusarium* populations and toxin accumulation in different cereals. Chen et al. [74] concluded that NX occurrence on barley, wheat, and other cereals is often correlated to climatic conditions in different geographic locations. Thus, the knowledge of the predominance of chemotypes under expected climatic conditions and specific hosts is relevant in developing appropriate surveillance programs.

Although *Fusarium poae* is regarded as a minor pathogen within the FHB complex, the wide variety of mycotoxins it can synthesize, including trichothecenes, enniatins, and BEA, should make this species of great concern [7]. Our data showed that *F. poae* was able to produce NIV, DAS, NEO, MAS, T-2 tetraol, BEA, aurofusarin, butenolide, and gybepyrone D. Barley is a primary source of some of these toxins in food and humans are exposed to them either through direct grain consumption or indirectly, by consuming mycotoxin-contaminated products such as beer. In a previous study NIV, NEO, enniatins (ENNs), BEA, T-2, and HT-2 toxins were evaluated during the malting process and a decrease in all mycotoxins was detected during steeping. However, a more than 500% increase in the germination stage was observed [75].

In the current research, lower DON contamination levels were determined in barley grains in comparison to those detected in a previous survey carried out in Argentina where contamination with mean levels of 2.36 mg/kg was reported [57]. In Brazil, barley samples were analyzed for DON contamination and this toxin was also detected in high levels ranging from 0.05 to 2.1 mg/kg [76]. In addition, 90% of the samples tested in Uruguay revealed the presence of DON, with levels ranging from the quantification limit to 3.74 mg/kg [77]. As regards NIV occurrence, few studies have analyzed this mycotoxin in barley grain samples from South America. Nogueira et al. [57] detected high DON contamination levels in Argentinian samples but, in agreement with the present study, only a few samples were contaminated with NIV. However, although DON and NIV contamination detected in barley grains was lower in comparison with other reports, the incidence of species with the potential to produce a wide variety of mycotoxins could be a considerable risk factor for their production if meteorological conditions favor their production. In this sense, Habschied et al. [78] demonstrated the effect of agro-climatic conditions on multi-mycotoxin occurrence, evaluating different winter barley genotypes during a 3-year assay.

The control of trichothecenes in barley grains is essential for the malting and brewing industries, as these are the primary mycotoxins that accumulate in barley infected by *Fusarium* species [79,80]. Before the micro-malting process, if barley grains are infected with *F. graminearum* ss and/or contaminated with DON, additional applications of the biocontrol agent can be used during different stages of this process. The results of the real-time PCR analysis showed that the most effective biocontrol on *F. graminearum* ss was observed at the end of the process when the biocontrol agent was applied before the germination stage. Possibly *F. graminearum* ss continues to grow in the absence of *B. velezensis* RC218, since under its biocontrol effect its growth was limited. In an in vitro study using a barley-based medium, *Bacillus velezensis* RC218 demonstrated the ability to control *Fusarium graminearum* sensu stricto at a distance [53]. More recently, it was shown that *B. velezensis* RC218 produces several antifungal lipopeptides, including iturins, surfactins, bacillomicins, mycosubtilins, and fengycins, among others [81]. Additionally, volatile compounds produced by this strain have proven effective in inhibiting the growth of *F. graminearum* (personal communication). Conversely, in the micro-malting experiments, DON decrease was observed at the end of the steeping stage in both *F. graminearum* ss treatments when the biocontrol agent was applied at the beginning of the steeping stage, but not when it was applied at the beginning of the germination stage. Lower DON concentration at the end of steeping in contaminated barley grains may be attributable to the dilution effect or its transformation into other metabolites. Maul et al. [82] observed that the first 17 h of steeping and later germination induce the glycosylation of DON to DON-3-Glc, which explains the natural decrease in DON concentration during the malting process.

## 4. Conclusions

The findings of this study highlight the prevalence of *Fusarium* species in barley grains, with *Fusarium graminearum* sensu stricto identified as the most common species across the regions studied. Although *Fusarium poae* was primarily isolated in the Ferré region, its presence is important due to the wide range of mycotoxins it can produce. Consequently, monitoring *Fusarium* contamination is essential, particularly considering regional, genotypic, and seasonal variations. While the levels of deoxynivalenol and nivalenol in the grains were generally low, the presence of mycotoxin-producing species still poses potential risks to food safety and grain quality, as environmental conditions could favor their production.

The biocontrol was not as effective in the toxin treatment as it was in the fungal inoculum treatment at the end of the micro-malting process. Therefore, the biocontrol agent could only be affecting fungal growth. The implementation of this biocontrol strategy at the malting stage, added to the efforts intended to develop alternatives used at the field level, could decrease the potential risk of deoxynivalenol contamination in beer as the final product. This would lead to the improvement of the quality and safety of beer made from barley grains in Argentina.

## 5. Materials and Methods

### 5.1. Isolation of Fusarium Species, Determination of Their Chemotypes and Mycotoxin Natural Incidence on Barley from Argentina’s Growing Regions

#### 5.1.1. Barley Sampling at Harvest Stage

**Figure 4:**
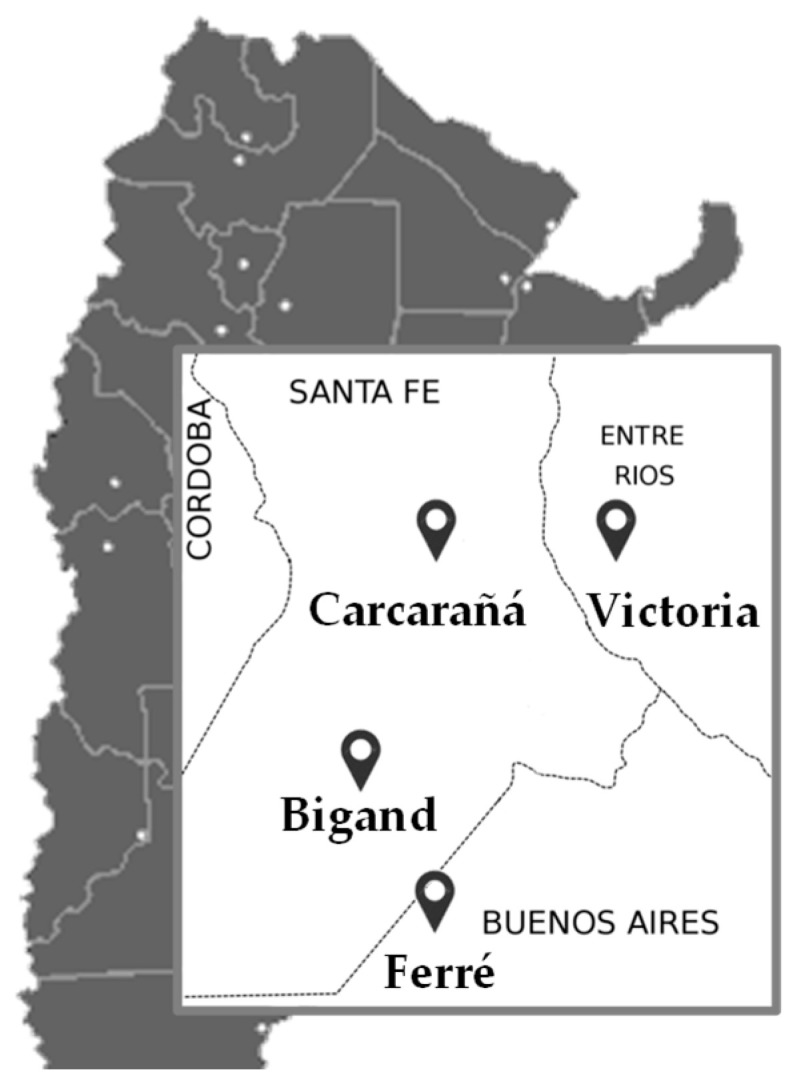
Argentinian barley-growing regions sampled.

Fifty-six samples of barley grains of different varieties (MP1012, Danielle, Montoya, Andreia, Shakira, Voyager, and MP2122) commonly used for the brewing process were analyzed. They were collected during the 2016 and 2017 harvest seasons from different locations in Argentina: Ferré (Buenos Aires province), Carcarañá (Santa Fe province), Bigand (Santa Fe province) and Victoria (Entre Ríos province) (Figure 4). Seven samples from each locality were analyzed for each year.

#### 5.1.2. *Fusarium* Isolation and Species Identification

From each barley sample, 300 grains were randomly selected, surface disinfected for 1 min in sodium hypochlorite solution (1%), rinsed three times in sterile distilled water, and 100 of those grains were placed on the surface of Nash–Snyder medium [83]. The plates were incubated at 25 °C for 7 days with periods of 12 h of white light and 12 h of black light. After incubation, the percentage of infected grains was determined and, from each sample, a representative number of *Fusarium* strains (the square root of the total number) were isolated. For morphological identification and perithecia production, the colonies were sub-cultured on Spezieller Nährstoffarmer Agar (SNA) [84] and on Carrot Agar, respectively, following the methodology described by Leslie and Summerell [83]. Molecular characterization at the species level of these strains was performed by sequencing the partial sequence of the elongation factor 1α (TEF-1 α), using the primers described by O’Donnell et al. [85,86].

#### 5.1.3. Chemotype Determination of *Fusarium graminearum* Sensu Stricto and *Fusarium poae* Strains

The *Fusarium* strains, identified as *Fusarium graminearum* sensu stricto and *Fusarium poae*, were cultured in Erlenmeyer flasks (250 mL) containing 25 g of long-grain rice. A total of 10 mL of distilled water was added before autoclaving for 30 min at 121 °C, twice. Each flask was inoculated with a 3 mm diameter agar disk taken from the margin of a colony grown on SNA at 25 °C for 7 days. Flasks were shaken once a day by hand for 1 week. These cultures were incubated for 28 days at 25 °C in darkness. At the end of the incubation period, the contents of the flasks were dried at 50 °C for 24 h and then stored at 20 °C. Toxin analysis was carried out according to Sulyok et al. [87] with some modifications. Each sample was finely ground in a laboratory grinder and homogenized. A sub-sample of ground rice (1 g) was extracted by mixing with 4 mL acetonitrile:water:acetic acid (79:20:1, *v*/*v*/*v*) and shaken for 2 h on an oscillatory shaker. Then, the mixture was centrifuged at 3000 rpm for 2 min and 4 mL of the supernatant evaporated to dryness with N_2_. Detection and quantification of mycotoxins were performed using a QTrap 4000 mass spectrometer (Applied Biosystems, Foster City, CA, USA) equipped with a Turbo-Ion-Spray (LC/ESI-MS/MS) electrospray ionization source, according to Malachová et al. [88].

#### 5.1.4. Deoxynivalenol and Nivalenol Analysis in Barley Grains

In addition to *Fusarium* strain identification in barley samples, the natural incidence of DON and NIV contamination was determined in the same samples. The mycotoxin analysis was performed using the method described by Chiotta et al. [89] with some modifications. Grains were finely ground in a laboratory grinder and homogenized. A subsample (25 g) was extracted by mixing with 100 mL acetonitrile:water (84:16, *v*/*v*), shaken for 30 min in an oscillatory shaker, and then filtered through N° 4 Whatman filter paper. Clean-up was carried out using a Mycosep 227 column (Romer Labs Inc., Austria). The filtrate (8 mL) was transferred to a culture tube and slowly pressed into the interior of the tube with the rubber flange end turned down until 6 mL of the extract had passed through the column. Then, 4 mL of the purified extract was transferred to a vial and evaporated to dryness with N_2_ at 60 °C. The dried residue was re-dissolved in 400 mL water:methanol (88:12, *v*/*v*), homogenized in a vortex mixer, and injected into the HPLC system (Waters e2695 separations module, Milford, MA, USA). Chromatographic separations were carried out using a stainless steel C18 reversed-phase column (150 mm × 4.6 mm, 5 μm particle size; Luna-Phenomenex, Torrance, CA, USA) coupled with a pre-column (20 mm × 4.6 mm, 5 μm particle size; Luna-Phenomenex, Torrance, CA, USA). Mycotoxins were detected at 220 nm using a Waters 2998 diode array detector (Waters Corporation, Milford, MA, USA). The mobile phase consisted of water:methanol (88:12, *v*/*v*) at a flow rate of 1.5 mL/min. The detection limit (LD) for both toxins was 0.01 mg/kg, determined based on a signal-to-noise ratio of 3:1. The retention times for NIV and DON were 4.6 min and 10.1 min, respectively.

### 5.2. Biocontrol Assay During the Micro-Malting Process

Based on the previous results, which showed that *Fusarium graminearum* ss is frequently isolated from barley, a biocontrol study was conducted on the micro-malting process, with a particular focus on this species and DON production.

#### 5.2.1. Biocontrol Agent Inoculum

*Bacillus velezensis* RC218 was originally isolated from wheat anthers during the 2004 harvest season [48]. It was selected as a biocontrol agent due to its ability to inhibit the growth of *Fusarium graminearum* ss and *Fusarium poae*, as well as reduce DON contamination under in vitro, greenhouse, and field conditions [53]. The bacteria were cultured in a basal medium containing 10 g/L sucrose and 5 g/L yeast extract, and incubated for 48 h at 28 °C with shaking at 150 rpm. Following this, the strains were subcultured into fresh liquid medium modified with NaCl (a_w_ 0.97) to physiologically enhance the strains by promoting the intracellular accumulation of betaine [90]. After 24 h growth at 28 °C and 150 rpm, two replicates of culture serial dilutions were plated in nutrient agar and incubated at 28 °C for 24 h. The number of colony-forming units (cfu) was determined by serial dilution and plate counting technique in the agarised basal medium (2% agar). The biocontrol agent inoculum was adjusted to 10^6^ cells/mL using the water using the water employed in the assays described below.

#### 5.2.2. Micro-Malting

The micro-malting process was conducted using barley samples of the Andreia genotype. They were evaluated in three different assays: artificially infected grains with *F. graminearum* ss in field trials (control ++), naturally infected grains with *F. graminearum* ss (control +), and naturally contaminated grains with high levels of DON (toxin +). The artificially infected grains were obtained from a previous field assay [53] where barley spikes were inoculated with *F. graminearum* ss in order to promote a high infection. For the biocontrol treatments, *Bacillus velezensis* RC218 was applied at two stages during the process: (1) before the steeping stage (treatment 1), and (2) when the moisture adjustment was carried out, prior to the germination stage (treatment 2) (Table 5). The biocontrol application was performed at each stage at a concentration of 10^6^ cells/mL. The micro-malting experiment was carried out on a pilot scale. Thus, 300 g of grains were steeped with 500 mL of water at room temperature with two alternate stages of steeping (5 h each) and two alternate periods of air rest for a total of approximately 18 h. The O_2_ content in the steeped water was manually applied every 1 h and the CO_2_ content was extracted by placing the vessels in a microgrid unit (Seeger, C. Seeger, Maschinenfabrik, Stuttgart, Germany). For the germination process, the grain moisture was adjusted to 47%, and barley grains were allowed to germinate for 4 days at 18 °C. For the kilning process, the temperature was increased from 55 °C to 82 °C. The kiln malt was allowed to cool and the culms were removed.

The malt obtained from each treatment was evaluated by analyzing yield losses due to malting, β-glucans, diastatic power, nitrogen, and protein content (this analysis was carried out by Cervecería y Maltería Quilmes).

#### 5.2.3. *Fusarium graminearum* Sensu Stricto DNA Quantification by Real-Time PCR and Mycotoxin Determination

Samples for *F. graminearum* ss DNA quantification by real-time PCR and DON determination were collected at the end of steeping, germination, and kilning stages. Grains of each treatment were milled and total DNA was extracted from approximately 10 mg of the pulverized subsamples using the DNeasy 96 plant kit (QIAGEN, Hilden, Germany) with some modifications. The incubation of the mixture composed of substrate, Buffer AP1, and RNase A mixture was performed for 30 min instead of 10 min, as the original method describes. Real-time quantifications were monitored on an ABI Prism7500 Sequence Detection System (Applied Biosystems, Foster City, CA, USA). TaqMan reactions were performed in a 25 μL reaction mixture containing 12.5 μL of TaqMan 2X universal PCR master mix (Applied Biosystems), 100 nmol/L of FAM-labelled probe and internal control probe, and 400 nmol/L of forward and reverse primer for both the target *F. graminearum* ss [91,92] and the internal positive control [93]. qPCR reactions were performed on 2 μL of DNA preparations from barley samples and pure pathogen DNA. Thermal cycling conditions consisted of 10 min at 95 °C, followed by 40 cycles of 95 °C for 15 s and 60 °C for 1 min. A standard curve was generated by using 10-fold serial dilutions of pure DNA in the range of 0.1 to 1 × 10^4^ pg/μL, and five replicates of pathogen quantifications were carried out. Pathogen DNA samples were run in parallel using a 10-fold serial dilution ranging from 0.1 to 1 × 10^4^ pg/μL, as reference. Based on the CT values obtained with qPCR for the different pathogen DNA dilution series and for DNA extracts from debris samples, the DNA concentrations of pathogen in the samples were calculated and expressed as picograms (pg) of fungal pathogen DNA per mg of plant tissue (dry weight). Robustness of the qPCR quantifications was analyzed according to Waalwijk et al. [92] and Chiotta et al. [89] using the same primers and probes. Deoxynivalenol determination was performed as it was described in Section 5.1.4.

### 5.3. Data Analyses

Data on fungal and mycotoxin determination were evaluated by analysis of variance ANOVA test, followed by Tukey mean separation test (*p*-value < 0.001, *p*-value < 0.05). Statistical analyses were performed using InfoStat version 2018p software [94] and SigmaStat version 3.5 (SPSS, Chicago, IL, USA) programs.

## Figures and Tables

**Table 1:** In vitro biosynthesis of secondary metabolites by *Fusarium graminearum* sensu stricto strains.

Mycotoxins	Range (mg/kg) ^a^	Mean (mg/kg) ^b^
**Trichothecene type B**		
Deoxynivalenol (DON)	3.9–4409.4	2973.9
15-Acetyldeoxynivalenol (15-ADON)	50.2–483.5	207.4
3-Acetyldeoxynivalenol (3-ADON)	0.4–573.4	329.3
Nivalenol (NIV)	1.2–17.2	9.8
**Trichothecene type A**		
NX-2	113.8–459.7	286.7
NX-3	19.0–50.2	354.1
**Zearalenone and derivatives**		
Zearalenone (ZEA)	3.0–264.7	86.4
Zearalenone-sulfate (ZEA-S)	20.6–528.0	219.5
Alpha-Zearalenol (α-ZEA)	0.4–0.7	0.5
Beta-Zearalenol (β-ZEA)	0.3–1.9	1.2
**Others**		
Sambucinol	43.3–156.4	108.8
Culmorin	9.8–1448.4	312.3
15-hydroxyculmorin	3.7–2510.6	830.0
5-hydroxyculmorin	94.5–1568.0	874.1
Aurofusarin	1.2–1379.8	158.7
Butenolid	1.1–15.8	5.3
Gibepyron D	1.8–159.1	72.8

^a^ Mycotoxin level ranges obtained, ^b^ Mean levels of mycotoxins produced in grain rice.

**Table 2:** In vitro biosynthesis of secondary metabolites by *Fusarium poae* strains.

Mycotoxins	Range (mg/kg) ^a^	Mean (mg/kg) ^b^
**Trichothecene type B**		
Nivalenol (NIV)	0.3–377.2	65.9
**Trichothecene type A**		
Diacetoxyscirpenol (DAS)	0.1–12.9	1.2
Neosolaniol (NEO)	0.1–14.5	0.8
Monoacetoxyscirpenol (MAS)	0.1–28.6	2.7
T-2 Teatrol (T_2_)	0.1–74.1	4.3
**Depsipeptides**		
Beauvericin (BEA)	4.4–68.1	46.5
**Others**		
Aurofusarin (AUF)	0.25–663.5	96.3
Butenolide	0.9–67.0	4.9
Gibepyrone D	1.1–59.7	8.1

^a^ Mycotoxin level ranges obtained, ^b^ Mean levels of mycotoxins produced in grain rice.

**Table 3:** Natural occurrence of deoxynivalenol and nivalenol in barley grains.

Season	Location	Deoxynivalenol			Nivalenol		
		Positive Samples/Total ^b^	Range ^c^ (mg/kg) ^a^	Mean ^d^ (mg/kg) ^a^	Positive Samples/Total ^b^	Range ^c^ (mg/kg) ^a^	Mean ^d^ (mg/kg) ^a^
**2016**	**Ferré**	7/7	0.18–0.55	0.34	7/7	0.25–1.00	0.62
	**Carcarañá**	7/7	0.46–1.77	1.00	1/7	0.55	0.55
	**Bigand**	13/13	0.10–0.51	0.30	3/13	0.12–0.50	0.37
	**Victoria**	9/11	0.10–0.54	0.20	0/11	DL	-
**2017**	**Ferré**	7/7	0.5–1.00	0.67	5/7	0.62–0.94	0.89
	**Carcarañá**	6/7	0.2–0.60	0.42	1/7	0.70	0.70
	**Bigand**	2/7	0.1–0.30	0.15	0/7	DL	-
	**Victoria**	0/7	DL	-	0/7	DL	-

DL: Detection limit: 0.01 mg/kg. ^a^ ppm: mg/Kg of DON and NIV detected in barley samples. ^b^ Samples contaminated with DON or NIV of total. ^c^ DON or NIV level ranges obtained. ^d^ Mean levels of DON or NIV detected in barley.

**Table 4:** Quality parameters of the barley samples obtained during the micro-malting process.

Trials	Quality Parameters					
	H (%)	F (min)	ME (%)	ADF (%)	FAN (mg/100 g)	β-Glucans (mg/L)
**Control (+)**	7.1	23	81.1	80.6	187	59
Steeping application (T_1_)	7.1	23	81.0	79.5	179	76
Germination application (T_2_)	7.1	20	81.3	81.0	178	70
**Control (++)**	7.1	20	82.0	79.7	176	65
Steeping application (T_1_)	7.1	20	81.9	80.7	169	75
Germination application (T_2_)	6.9	25	81.8	79.6	165	67
**Toxina (+)**	6.9	25	81.3	82.3	199	78
Steeping application (T_1_)	7.1	30	81.4	83.4	194	66
Germination application (T_2_)	7.2	20	81.2	82.9	202	73

H: humidity. F: filtration. ADF: Apparent degree of fermentation. ME: malt extract. FAN: free amino nitrogen.

**Table 5:** Treatments applied during micro-malting process.

Assay	Treatments	
Control (++)	Artificially infected grains with *F. graminearum* ss in field trials	
	T_1_	*B. velezensis* RC218 applied before the steeping stage
	T_2_	*B. velezensis* RC218 applied prior to the germination stage
Control (+)	Naturally infected grains with *F. graminearum* ss	
	T_1_	*B. velezensis* RC218 applied before the steeping stage
	T_2_	*B. velezensis* RC218 applied prior to the germination stage
Toxin (+)	Naturally contaminated grains with high levels of DON	
	T_1_	*B. velezensis* RC218 applied before the steeping stage
	T_2_	*B. velezensis* RC218 applied prior to the germination stage

## Data Availability

The original contributions presented in this study are included in the article. Further inquiries can be directed to the corresponding authors.
